# Retrospective observational study of the association of peak blood glucose during the second 24 hours of admission with hospital-acquired complications in non-critical care admissions to a tertiary referral teaching hospital

**DOI:** 10.1136/bmjopen-2024-089652

**Published:** 2025-01-14

**Authors:** Barbara Depczynski, Getiye Dejenu Kibret, Andrew Georgiou, Sue Mei Lau

**Affiliations:** 1 Department of Diabetes and Endocrinology, Prince of Wales Hospital and Community Health Services, Randwick, New South Wales, Australia; 2 UNSW, Sydney, New South Wales, Australia; 3 Macquarie University, Sydney, New South Wales, Australia

**Keywords:** Hospitalization, Diabetes & Endocrinology, General diabetes

## Abstract

**Abstract:**

**Introduction:**

Stress hyperglycaemia at hospital presentation is associated with poorer outcomes. Less is known about the risk of poorer outcomes according to achieved glycaemia early in the admission.

**Research design/methods:**

This was a retrospective observational study of patients admitted to non-critical care wards. The aim was to determine the relationship between the day 2 peak blood glucose and the occurrence of hospital-acquired complications (HACs) or in-hospital mortality. A Cox proportional hazards model, adjusted for relevant covariates, was used to evaluate the impact of day 2 peak glucose on HACs and in-hospital mortality, and we identified peak glucose thresholds correlating with an increase in risk.

**Results:**

For the whole cohort, day 2 peak glucose was associated with an increased risk of any HAC, aHR=1.06, 95% CI: 1.04, 1.07; but not in-hospital mortality, aHR=0.98, 95% CI: 0.94, 1.01. The risk of HAC infection increased by 4.6% for every mmol/L rise in day 2 peak glucose (aHR=1.05, 95% CI: 1.02, 1.08) in the diabetes cohort compared with 5.5% (aHR=1.06, 95% CI: 1.00, 1.11) in the non-diabetes cohort. The risk of HAC cardiac in the diabetes cohort increased by 5.3% (aHR=1.05, CI: 1.01, 1.10) per mmol/L increase in day 2 peak glucose; no association was found in the non-diabetes cohort (aHR=1.03, 95% CI: 0.94, 1.13). The risk for in-hospital mortality was associated with day 2 peak glucose, aHR=1.11, 95% CI: 1.03, 1.20, in patients without diabetes, but not in patients with diabetes, aHR=1.00, 95% CI: 0.95, 1.06. There was an increase in the risk of HAC once day 2 peak blood glucose exceeded 19.0 mmol/L (whole cohort), with thresholds of 13.6 mmol/L in the non-diabetes group and 19.5 mmol/L in the diabetes group.

**Conclusion:**

The peak glucose on day 2 was a predictor of HAC in the entire cohort and in-hospital mortality in patients without diabetes.

**STRENGTHS AND LIMITATIONS OF THIS STUDY**

- Our findings are from real-world data, derived from a large number of heterogeneous admissions.
- We ensured that there was a temporal separation between the occurrence of hyperglycaemia and any hospital-acquired outcomes.
- A limitation of this study is that the data are retrospective and observational.
- The analysis was limited to point-of-care glucose testing, rather than more granular sources such as subcutaneous glucose levels.

## Introduction

 Diabetes and hyperglycaemia are major risk factors for adverse events during hospital admission.^1 2^ Optimal early blood glucose (BG) targets on non-intensive care unit (ICU) wards are not well delineated; current recommendations for therapeutic intervention at 10 mmol/L for hospitalised patients without critical illness have been extrapolated from studies in ICU settings.^1^ Current guidelines from the American Diabetes Association recommend for the management of hyperglycaemia in non-critical care settings, a glycaemic goal of 5.6–10.0 mmol/L for most hospitalised patients, whether it is new hyperglycaemia (newly diagnosed diabetes or stress hyperglycaemia), or hyperglycaemia related to diabetes prior to admission.^3^ The Joint British Diabetes Societies for Inpatient Care group has recommended a blood glucose target range of 6.0–10.0 mmol/L for most inpatients with hyperglycaemia, with an acceptable range of 4.0–12.0 mmol/L, with modification of lower acceptable glucose according to whether a patient is prescribed insulin or insulin secretagogues.^2^

In assessing glycaemia and hospital outcomes, many studies have focused on admission glycaemia alone^4^ or whole-of-admission mean glycaemia.^5^,^7^ Hyperglycaemia within the first 24 hours of admission is likely a reflection of stress hyperglycaemia and prior glycaemic control.^8 9^ Whole-of-admission glycaemia may be influenced by the bidirectional relationship between glycaemia and adverse hospital events.^10^ Assessment of day 2 glycaemia may provide a truer indicator of the quality of diabetes care and associated benefits,^11 12^ although we acknowledge that the glucose threshold for adverse outcomes does not necessarily determine the threshold for insulin intervention in a hospital setting. In intensive care units (ICU), earlier achievement of glycaemic control is associated with better outcomes.^13 14^ In patients admitted for COVID-19 infection, hyperglycaemia after admission (but not admission hyperglycaemia) was a strong predictor of death among non-ICU patients.^12^ Similarly, mean glycaemia during the first 48 hours of admission for acute myocardial infarction was a better predictor of mortality than admission glucose.^15^ To date, however, a detailed understanding of the association of day 2 glycaemia with hospital outcomes in a heterogeneous group of medical or surgical patients admitted to non-ICU wards is lacking.

### Aim

To determine the relationship between the peak BG observed during day 2 of admission and the subsequent occurrence of hospital-acquired complications (HACs) or in-hospital mortality in patients with and without diabetes on non-ICU wards. We also aimed to determine whether there is a glucose threshold above which HACs or in-hospital mortality are more likely to occur, according to the presence or absence of diabetes. A secondary aim was to compare the association of peak glucose levels on day 1 and day 2 with the risk of HAC.

## Methods

### Setting

Our hospital is a tertiary referral teaching hospital in an urban area of Australia. Inpatient care of diabetes is primarily the responsibility of the admitting team staff. Consultations to the diabetes service are made on an ad hoc basis by formal referral. There are formal policies for the management of hypoglycaemia and perioperative care of patients with diabetes and hyperglycaemia. The use of non-insulin antihyperglycaemic therapies isnot prohibited, and there is no standing order to discontinue non-insulin antihyperglycaemic agents and routinely prescribe a basal-bolus insulin regimen during the admission. Hospital protocols mandate regular point-of-care (POC) blood glucose (BG) testing for those with known diabetes (pre-meal and bedtime if eating; sixth hourly at least if nil by mouth). Opportunistic POC BG testing is encouraged for those without known diabetes at risk for hyperglycaemia, for example, patients on stroke and cardiac wards, or those on glucocorticoids.

### Participants

Inclusion criteria included age ≥18 years and a length of stay (LOS) >48 hours, admitted under an acute medical or surgical team to a non-ICU ward, between 01/01/2018 and 28/02/2021. We excluded pregnant patients or those with no POC BG data for the first and second 24-hour periods of their hospital admission, or where a HAC or death occurred during the first 48 hours of hospital admission. Patients admitted for the management of a hyperglycaemic or hypoglycaemic emergency were also excluded. Where more than one admission occurred during the study period, only the first admission was included in the analysis.

### Definitions

Diabetes was defined as documented in the medical record and included patients with pre-existing or a new diagnosis of diabetes. POC BG metres (StatStrip, Australasian Medical and Scientific (AMSL)) are used in ward areas. HbA1c results (within 6 months of admission) were documented in the medical record. HACs were identified according to the criteria defined by the Australian Commission on Quality and Safety in Healthcare.^16^ The HAC composite endpoint included pressure injury, falls resulting in fracture or intracranial injury, healthcare-associated infection (HAC infection) (including urinary tract infection (UTI), pneumonia, bacteraemia, surgical site infection, cannula infection, multiresistance organism on any culture, infection of an implanted device or gastrointestinal (GIT) infection), surgical complications requiring unplanned return to theatre, respiratory complications, venous thromboembolism (VTE), acute renal failure (ARF) (defined as a new need for dialysis during admission), gastrointestinal bleeding, delirium, incontinence or cardiac complications (HAC cardiac) (including heart failure, arrhythmia, STEMI or NSTEMI). The Charlson comorbidity index (CCI) was calculated for each admitted patient admission based on the ICD-10 codes from previous admissions to facilities within the health district, excluding the present admission, to represent the index at the point of that admission, and the scores for diabetes were omitted.^17^ We then categorised the CCI scores, classifying a score of 0 as none, a score of 1–2 as mild, a score of 3–4 as moderate and a score of 5 or higher as severe. The eGFR was calculated using the CKD-EPI creatinine equation.^18^

### Data extraction

Patient information, clinical outcomes, and point-of-care (POC) capillary BG values recorded during admission and captured in the electronic medical record (Cerner Millennium) and inpatient administration database were retrieved and used for this analysis. We extracted epidemiological information (age, gender, length of stay, admitting team, type of admission—whether medical or surgical team and via emergency or as a booked admission). The inpatient administrative database manages the demographic data, discharge diagnostic codes, and separation data. Fidelity between coding and the medical file was confirmed by manual review of all files, which were compared with ICD-10 codes generated by the trained medical coders and submitted to the hospital administrative database.

### Outcome measurements

The primary endpoint was the occurrence of any HAC (HAC composite) and in-hospital mortality. Other endpoints examined were HAC infection and HAC cardiac. Additionally, we compared the predictive value of peak BG in the first 24 hours compared with the second 24 hours of hospitalisation on hospital-acquired adverse outcomes.

Ethics approval was granted by South Eastern Sydney Local Health District Human Research Ethics Committee (2020/STE03529) who waived the need for consent. The study adhered to guidelines for strengthening the reporting of observational studies.^19^ This work was supported by funding from a National Health and Medical Research Council (NHMRC) Partnership Project 2 006 755.

### Patient and public involvement

Patients and the public were not involved in the design, or conduct, or reporting, or dissemination plans of our research.

### Statistical analysis

Descriptive data were summarised using numbers with percentages, means with SD and medians with first and third quartiles when appropriate. The Kaplan–Meier (KM) survival function was used to plot survival curves for each outcome cohort. A Cox proportional hazards (PH) model was used to evaluate the impact of peak glucose level in the first and second 24 hours of admission on hospital-acquired adverse outcomes. All the Cox regression models were adjusted for age, gender, admission type (emergency vs elective), admitting team (medical vs surgical), CCI, admission eGFR, prescription of oral or intravenous glucocorticoids during the first 24 hours of admission and year of admission. We capped the follow-up period at 60 days as a directly causal relationship between day 2 glucose and outcomes beyond 60 days was thought unlikely and to avoid skewing of the outcomes by the few individuals with a prolonged hospital admission; patients who did not experience any outcomes within this time frame were considered censored. HRs with corresponding 95% CI were presented, and a p value of 0.05 was considered statistically significant.

We further estimated the adjusted HRs (aHR) at specific peak glucose levels in the second 24 hours of admission, with a focus on identifying the peak glucose threshold that correlates with an increase in risk. The relationships of peak glucose levels with the outcomes of interest were further visualised through HR plots.

PH assumptions for categorical variables were checked using graphical (log-log survival curves) and statistical (Schoenfeld residuals test) methods. For the continuous variables, PH assumptions were evaluated by incorporating an interaction term between the variable and a function of time into the Cox model, and a significant coefficient for the interaction term suggests a violation of the PH assumption.

We examined how day 1 and day 2 peak glucose levels affect HACs by fitting these variables into separate models while controlling for the same set of confounders in both analyses. We used aHRs and the Akaike information criterion (AIC) to compare the influence of peak glucose levels in the two different time periods. Both metrics would allow for direct comparison, with the HRs comparing effect sizes and the AIC determining which period’s peak glucose level best fits the model. All analyses were conducted using R software V.4.3.2.^20^

## Results

### Demographic and clinical characteristics

After applying exclusion criteria, a total of 6967 patients were included in the study (online supplemental figure 1). Baseline characteristics, according to diabetes status or quartiles of day 2 peak glucose, are shown in table 1. Of the whole cohort, 3930 (56.4%) were men and 3587 (51.5%) had a diagnosis of diabetes (type 1 in 61 patients). The median (IQR) age of participants was 71 (59–80) years, and 5295 (76%) were an emergency admission. The mean (SD) number of BG measurements per person during day 2 was 3.3 (2.3) in the overall group with 4.2 (2.4) in the diabetes patients and 2.24 (1.6) in the non-diabetes patients. The peak glucose level during day 1 and day 2 of the admission had a mean of 8.2 mmol/L (SD: 2.9) and 8 mmol/L (SD: 2.6), respectively (table 1).

**Table 1:** Demographic and clinical characteristics of patients according to the presence or absence of a diagnosis of diabetes; and quantiles of day 2 peak glucose level

Variable	All	Diabetes	No diabetes	≤6.7 mmol/L	6.8–8.3 mmol/L	8.4<11 mmol/L	≥11 mmol/L
Patients, n	6967	3587	3380	1840	1746	1672	1709
Gender, n (%)							
Female	3037 (43.6)	1463 (40.8)	1574 (46.6)	881 (47.9)	769 (44)	683 (40.8)	704 (41.2)
Male	3930 (56.4)	2124 (59.2)	1806 (53.4)	959 (52.1)	977 (56)	989 (59.2)	1005 (58.8)
Age, median (Q1, Q3)	71 (59, 80)	74 (65, 81)	67 (52, 79)	69 (52, 80)	71 (58.25, 80)	72 (62, 81)	73 (63, 81)
Admission type, n (%)							
Emergency	5295 (76)	2658 (74.1)	2637 (78)	1526 (82.9)	1300 (74.5)	1175 (70.3)	1294 (75.7)
Elective	1672 (24)	929 (25.9)	743 (22)	314 (17.1)	446 (25.5)	497 (29.7)	415 (24.3)
Admitting team, n (%)							
Medical	4171 (59.9)	2184 (60.9)	1987 (58.8)	1180 (64.1)	1016 (58.2)	943 (56.4)	1032 (60.4)
Surgical	2796 (40.1)	1403 (39.1)	1393 (41.2)	660 (35.9)	730 (41.8)	729 (43.6)	677 (39.6)
LOS, median (Q1, Q3)	6 (4, 12)	6 (4, 11)	6 (4, 12)	6 (3, 11)	6.5 (4, 13)	7 (4, 13)	6 (4, 12)
Hospital mortality, n (%)	228 (3.3)	89 (2.5)	139 (4.1)	56 (3)	62 (3.6)	64 (3.8)	46 (2.7)
HAC types, n (%)							
HAC any	930 (13.3)	492 (13.7)	438 (13)	146 (7.9)	228 (13.1)	257 (15.4)	299 (17.5)
HAC infection	493 (7.1)	259 (7.2)	234 (6.9)	59 (3.2)	116 (6.6)	150 (9)	168 (9.8)
HAC cardiac	208 (3)	127 (3.5)	81 (2.4)	27 (1.5)	47 (2.7)	47 (2.8)	87 (5.1)
Charlson index, n (%)							
None	4760 (68.3)	2252 (62.8)	2508 (74.2)	1347 (73.2)	1223 (70)	1135 (67.9)	1055 (61.7)
Mild	1388 (19.9)	785 (21.9)	603 (17.8)	329 (17.9)	344 (19.7)	345 (20.6)	370 (21.7)
Moderate	508 (7.3)	351 (9.8)	157 (4.6)	91 (4.9)	111 (6.4)	125 (7.5)	181 (10.6)
Severe	311 (4.5)	199 (5.5)	112 (3.3)	73 (4)	68 (3.9)	67 (4)	103 (6)
Mean glucose day 1, mean (SD)	8.2 (2.9)	9.5 (3.3)	6.9 (1.6)	6.5 (1.3)	7.2 (1.6)	8.2 (2.1)	11.2 (3.5)
Mean glucose day 2, mean (SD)	8 (2.6)	9.1 (2.9)	6.9 (1.6)	5.7 (0.6)	6.9 (0.6)	8.3 (1)	11.5 (2.7)
HbA1c, mean (SD)n=number of patients with result	7.5 (2)	7.9 (2)n=1955	5.7 (0.6)n=492	5.8 (0.7)	6.3 (1.1)	7.2 (1.5)	8.6 (2.1)
DM therapies							
Insulin (with or without other antihyperglycaemic therapy)	838 (12)	838 (23.4)	0 (0)	6 (0.3)	59 (3.4)	187 (11.2)	586 (34.3)
Any non-insulin antihyperglycaemic therapy	2253 (32.3)	2253 (62.8)	0 (0)	271 (14.7)	496 (28.4)	680 (40.7)	806 (47.2)
No antihyperglycaemic therapy	3876 (55.6)	496 (13.8)	3380 (100)	1563 (84.9)	1191 (68.2)	805 (48.1)	317 (18.5)
Glucocorticoids during the first 24 hours	990 (14.2)	334 (9.3)	656 (19.4)	205 (11.1)	240 (13.7)	258 (15.4)	287 (16.8)
Admission eGFR, mean (SD)	73.3 (29.1)	66.5 (28.4)	80.6 (28.1)	78.4 (28.7)	75.8 (27.4)	71.7 (28.9)	66.9 (30)
Year, n (%)							
2018	2561 (36.8)	1474 (41.1)	1087 (32.2)	619 (33.6)	622 (35.6)	617 (36.9)	703 (41.1)
2019	2225 (31.9)	1108 (30.9)	1117 (33)	606 (32.9)	558 (32)	539 (32.2)	522 (30.5)
2020 and 2021	2181 (31.3)	1005 (28)	1176 (34.8)	615 (33.4)	566 (32.4)	516 (30.9)	484 (28.3)

n, number

### Incidence rates of HACs

The incidence rate for HAC composite was 16.2 per 1000 person-time, meaning that there were approximately 16.2 instances of HAC for every 1000 units of person-time observed. HAC infection had an incidence rate of 8.6 per 1000 person-time and cardiac-related HACs were observed at a rate of 3.6 per 1000 person-time. In-hospital mortality rate was 3.3 per 1000 person-time (table 2). The incidence rate of HAC composite, HAC infection and HAC cardiac was higher in patients with a formal diagnosis of diabetes compared with those without diabetes. Conversely, the incidence rate of in-hospital mortality was higher in the non-diabetes group (table 2).

**Table 2:** Incidence rates of hospital-acquired adverse outcomes per 1000 person-time, within 60 days of follow-up

Adverse outcome	Incidence rate (95% CI) per 1000 person-time			P value
	Overall incidence	Diabetes	No diabetes	
HAC any	16.2 (16.19, 16.30)	17.2 (17.10, 17.21)	15.3 (15.2, 15.31)	0.074
HAC infection	8.6 (8.58, 8.63)	9.05 (9.02, 9.09)	8.16 (8.12, 8.19)	0.266
HAC cardiac	3.63 (3.61, 3.65)	4.44 (4.41, 4.46)	2.82 (2.80, 2.84)	0.002
In-hospital mortality	3.33 (3.32, 3.35)	2.61 (2.59, 2.63)	4.05 (4.03, 4.07)	0.002

### Relationship between day 2 peak glucose level and HACs—whole cohort

**Figure 1:**
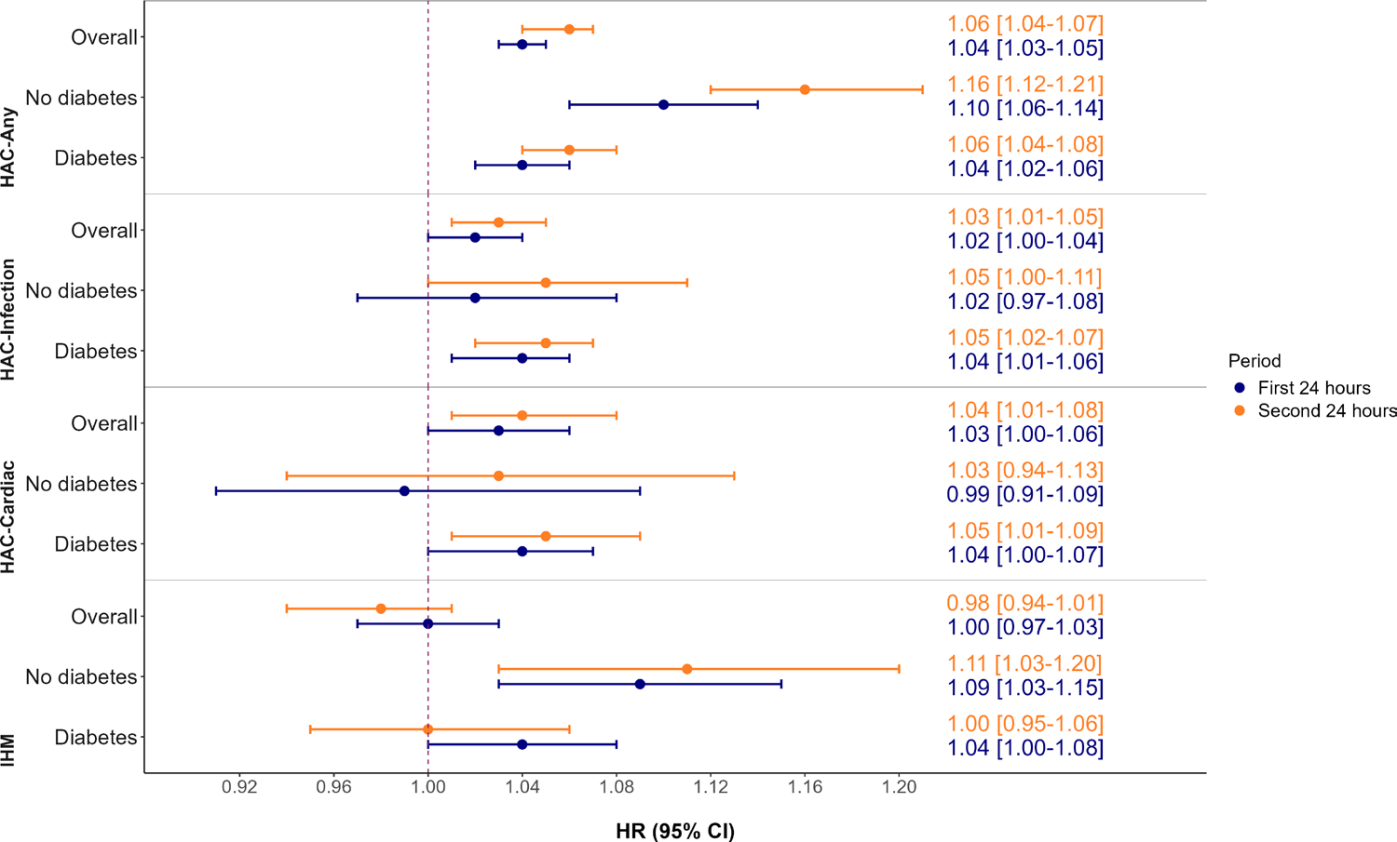
Relationship between the first and second 24 hours peak glucose level on hospital-acquired complications and in-hospital mortality. HRs for day 1 peak glucose are shown in blue, and HRs for day 2 peak glucose are shown in orange. IHM, in-hospital mortality.

We estimated the risks of HACs associated with peak blood glucose levels during the second 24 hours of admission, after controlling for age, sex, admission type (emergency vs elective), admitting team (medical vs surgical), CCI, admission eGFR, glucocorticoids during the first 24 hours and admission year. The risk of the HAC composite increased by 6.0% (adjusted HR (aHR) = 1.06, 95% CI: 1.04, 1.07) for every mmol/L rise in peak glucose levels over this time. The incidence of HAC infection increased by 3.0% (aHR=1.03, 95% CI: 1.01, 1.05) for each 1 mmol/L rise in peak glucose. Similarly, the risk of HAC cardiac increased by 4.4% (aHR=1.04, 95% CI: 1.01, 1.08) for each 1 mmol/L increase in peak blood glucose levels reported in the second 24 hours following admission. The in-hospital mortality risk was not significantly associated with the peak glucose level in the second 24 hours of admission (aHR=0.98, 95% CI: 0.94, 1.01) (figure 1).

We found high multicollinearity between day 1 and day 2 glucose levels, and so instead, we fitted separate models for each, controlling for confounders and compared the predictive power of the day 1 or day 2 peak glucose levels on HAC composite, HAC infection and HAC cardiac. The goodness-of-fit analysis, using aHRs and AIC parameters, revealed that day 2 peak glucose was a modestly better fit for the model than day 1 peak glucose (online supplemental table 1).

### Relationship between day 2 peak glucose level and HACs/in-hospital mortality, diabetes and non-diabetes cohorts

We separately estimated the risks of HACs and in-hospital mortality among patients with and without diabetes in relation to day 2 peak blood glucose levels, while adjusting for age, gender, admission type (emergency vs elective), admitting team (medical vs surgical), Charlson comorbidity index, admission eGFR, glucocorticoids during the first 24 hours and year of admission. The risk of HAC composite increased by 6% (aHR=1.06, 95% CI: 1.04, 1.08) in the diabetes cohort, and by 16% (aHR=1.16, 95% CI 1.12, 1.21) in the non-diabetes cohort, for every mmol/L increase in peak glucose levels. The incidence of HAC infection increased by 4.6% (aHR=1.05, 95% CI: 1.02, 1.08) in the diabetes cohort compared with 5.5% with marginal significance (p=0.051; aHR=1.06, 95% CI: 1.00, 1.11) in the non-diabetes cohort, for every mmol/L rise in day 2 peak glucose. The risk of HAC cardiac in patients with diabetes increased by 5.3% (aHR=1.05, 95% CI: 1.01, 1.10) for every mmol/L increase in day 2 peak glucose. In contrast, no significant association was found between day 2 peak glucose levels and HAC cardiac in patients without diabetes (aHR=1.03, 95% CI: 0.94, 1.13). The in-hospital mortality risk in patients without diabetes increased by 11% for every mmol/L increase in day 2 peak glucose (aHR=1.11, 95% CI: 1.03, 1.20); however, no association was observed in patients with diabetes (aHR=1.00, 95% CI: 0.95, 1.06) (figure 1).

### Threshold of risk for HAC, diabetes and non-diabetes cohorts

**Figure 2:**
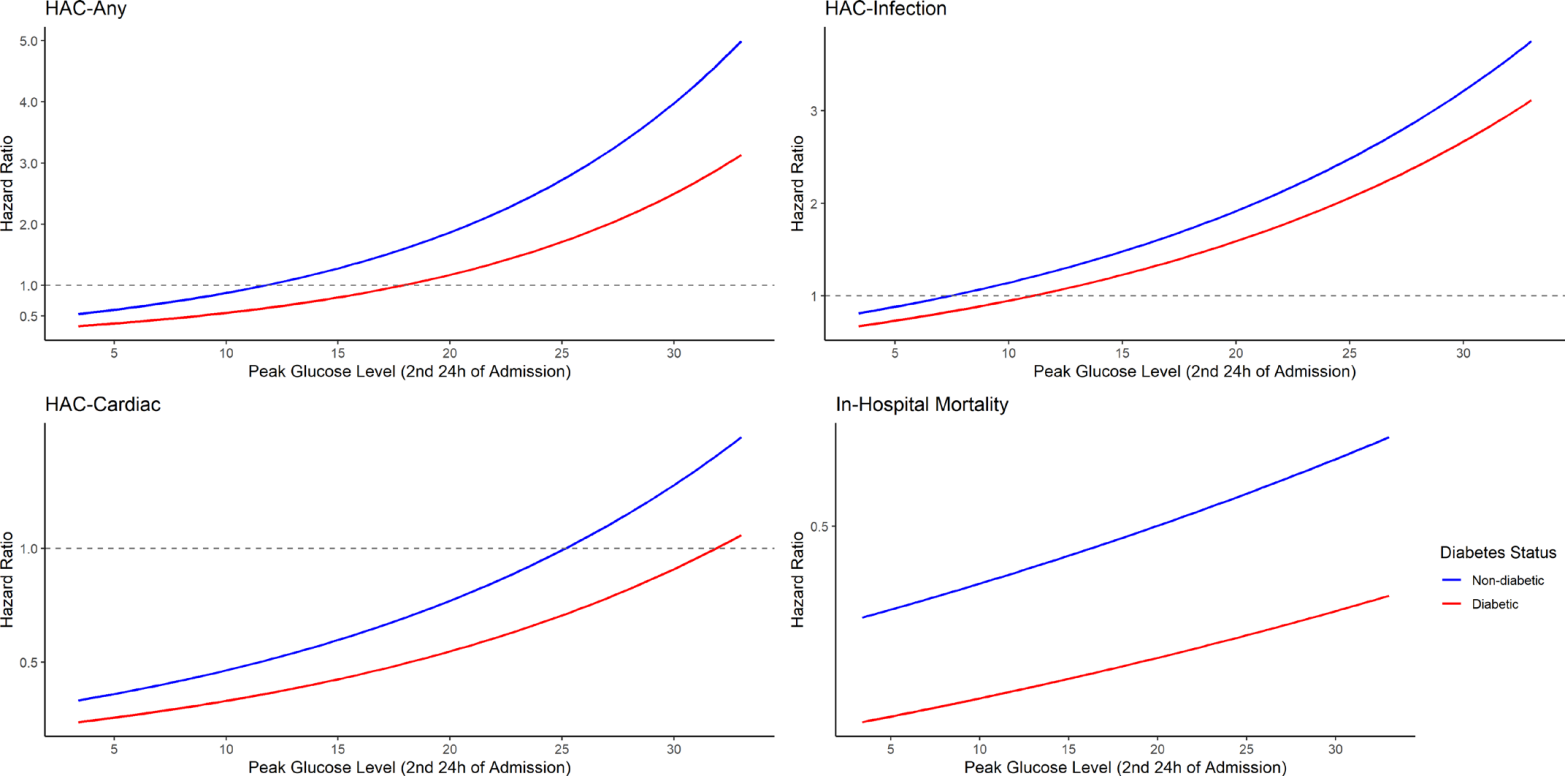
Risk of hospital-acquired complications (HACs) and in-hospital mortality as a function of day 2 peak glucose level after controlling for covariates (age, gender, admission type (emergency vs elective), admitting team (medical vs surgical), Charlson comorbidity index, glucocorticoid use first 24 hours and admission eGFR). The line of best fit is shown in blue for the non-diabetes cohort and in red for the diabetes cohort.

The relationship between day 2 peak glucose and HAC composite, HAC infection, HAC cardiac and in-hospital mortality are shown in figure 2. There was an incremental risk of HAC with increasing peak glucose; however, the risk of HAC composite rose more steeply in those without diabetes, so that for a given glucose the risk of HAC composite was higher in the non-diabetes group. There was an increased risk of HAC composite once peak blood glucose levels exceeded 19.0 mmol/L (whole cohort), with thresholds of 19.5 mmol/L in the diabetes group and 13.6 mmol/L in the non-diabetes group. There was an increased risk of HAC infection at a peak glucose of 12.9 mmol/L (whole cohort), with thresholds of 13.3 mmol/L for the diabetes group and 9.7 mmol/L for the non-diabetes group. There was an increased risk of HAC cardiac at a peak glucose of 24.9 mmol/L (whole cohort), with thresholds of 31.8 mmol/L in the diabetes group and 24.9 mmol/L in the non-diabetes group. The thresholds for in-hospital mortality could not be estimated with the available data range during the same period (figure 2).

## Discussion

In this retrospective cohort study, we evaluated the association of day 2 peak glucose with the occurrence of any HAC or in-hospital mortality. Our results demonstrate that for the whole cohort, day 2 peak glucose was associated with an increased risk for HAC composite, HAC infection and HAC cardiac, but not in-hospital mortality. The day 2 peak glucose predicted the occurrence of HACs better than day 1 peak glucose. The relationship between day 2 peak glucose and adverse outcomes differed between those with or without diabetes; however, it is apparent that for any day 2 peak glucose level, the outcome risk conferred was higher in the group without diabetes.

There are few studies assessing day 2 or early postadmission glucose levels and hospital outcomes. In patients admitted for COVID-19 infection, hyperglycaemia after admission, (but not admission hyperglycaemia) was a strong predictor of death among non-ICU patients.^12^ In critically ill trauma patients, the time to glucose normalisation was longer in those patients who died.^13^ In cardiac arrest patients, earlier achievement of euglycaemia was associated with higher in-hospital survival and better neurological outcomes.^14^ In the ICU setting, persistent hyperglycaemia in those without diabetes is associated with higher 90-day mortality as compared with patients without persistent hyperglycaemia.^21^ Among patients (with or without diabetes) admitted following myocardial infarction, a lower mean post-admission glucose level was associated with a lower risk of in-hospital mortality.^15^ Similarly, in people without diabetes, failure of glucose levels to decrease in the first 24 hours after admission with myocardial infarction predicted higher mortality.^22^

Day 2 glucose reflects impacts on glycaemic control after implementing measures to manage glycaemia and so provides a robust means to evaluate glycaemic targets to prevent HACs.^11 12^ This contrasts with admission glucose, which reflects stress hyperglycaemia and thus illness severity, as well as prior glycaemic control.^8 9^ Day 2 peak glucose may also reflect aberrant persistence of stress hyperglycaemia.^9^ The initial stress response to acute illness is a pro-inflammatory state that promotes hyperglycaemia; however, the resultant hyperglycaemia may, in turn, unfavourably modify subsequent immune responses.^23^

We found that the pattern of risk based on day 2 peak glucose differed between those with and without diabetes. In those with diabetes, hospital-acquired complications were increased, but not in-hospital mortality. In contrast, in those without diabetes, increasing day 2 peak glucose was associated with an increased risk of in-hospital mortality, HAC composite and HAC infection, but not HAC cardiac. The lack of a significant relationship between day 2 peak glucose and in-hospital mortality in diabetes patients is surprising, given the relationship between peak glucose and HAC composite, HAC infection and HAC cardiac. One explanation is that stress hyperglycaemia in patients without diabetes is a marker of illness severity, whereas hyperglycaemia in diabeticpatients also reflects other factors such as previous glucose control and in-hospital glycaemic management. It is well established that new hyperglycaemia in patients admitted to non-ICU wards is associated with increased mortality compared with patients with diabetes.^24^ Among emergency admissions, in patients without diabetes, there is a strong association between admission BG at presentation and in-patient mortality; however, in those with diabetes, a higher admission BG was not associated with increased in-hospital mortality.^4^ In admissions for acute coronary syndromes, a higher BG was associated with higher mortality in the non-diabetes group as compared with the diabetes group.^5^ A possible explanation for the association of day 2 peak glucose with HAC cardiac in diabetes patients only is that individuals with diabetes are likely to have a greater burden of underlying cardiovascular disease and may be more susceptible to the detrimental effects of catecholamine release^23^ that occurs in acute illness compared with those without diabetes. While day 2 peak glucose was associated with HAC infection risk in both diabetes and non-diabetic patients, the relationship was more significant in diabetic patients. A higher proportion of the non-diabetes patients were treated with glucocorticoids (19% vs 9.3% in the diabetes group), which would increase susceptibility to infection and potentially weaken the relationship between day 2 peak glucose and HAC infection. Hyperglycaemia impedes both innate immune response and adaptive immunity, which increases the risk of infection; however, in diabetes, the risk is further increased due to host factors^25^ and so may account for a higher risk in the diabetes group.

The threshold for onset of risk for HAC composite or HAC infection occurred at a lower day 2 peak glucose in patients without diabetes compared with those with diabetes. Several pathophysiological pathways may account for this finding. In patients with diabetes, chronic hyperglycaemia promotes cellular conditioning that may be protective against acute hyperglycaemia-mediated damage during critical illness.^8^ In contrast, stress hyperglycaemia in patients without prior diabetes is associated with greater inflammatory responses than those which occur with chronic hyperglycaemia in diabetes.^8^ Acute illness is associated with changes in fuel use, and metabolic responses may vary according to diabetes status and antihyperglycaemic therapy.^26^ For example, there is a differential impact of hyperlactataemia on the association between hyperglycaemia and hospital outcomes according to diabetes status.^27^ Induction of relative hypoglycaemia has been proposed to account for the failure of some intervention studies to demonstrate a benefit to lowering of glycaemia and of outcomes varying according to diabetes status.^28^ Thus, recent studies have explored personalised glucose targets that approximate pre-hospital glycaemic control.^29^

In our study, the likelihood of HAC infection rose at a peak glucose level of 12.7 mmol/L, with a threshold of 10.6 mmol/L for the non-diabetic group and 13.9 mmol/L for the diabetic group. These thresholds are similar to those used in our model of active surveillance via a virtual glucose management system, where identification of patients with a BG of at least 12 mmol/L, rather than sole reliance on ad hoc consultation, was associated with a reduction in hyperglycaemic patient-stay days and a decrease in hospital-acquired infection from 8.7% to 3.5% with an adjusted OR of 0.17 (95% CI: 0.05 to 0.61).^30^ The high BG threshold of risk for HAC cardiac in our cohort, compared with prior studies, may reflect differences in the underlying admission acuity.^31^ Similarly, for mortality, lower BG thresholds of 15.8 mmol/L (diabetes) and 11.3 mmol/L (non-diabetes) were previously reported in admissions for acute coronary syndromes^5^ rather than in a lower-risk general hospital cohort. We examined peak glucose as it has previously been shown that, among patients admitted with acute coronary syndromes or trauma, the peak glucose of the admission was associated with higher mortality,^5 32 33^ and peak glucose is an easier metric to assess on the wards. Derivation of mean glucose and SD of glucose from POC glucose tests is likely to be affected by the frequency of testing and may not be sufficiently granular to reveal associations between glucose excursions and outcomes.^34^ At the bedside, it is easier to implement management changes in response to a glucose threshold rather than a calculated mean glucose.

Our findings have implications for ward glycaemic management in terms of timing of intervention, BG targets and modification according to prior diabetes status. First, to achieve improved glycaemia by day 2, early intervention is required.^11^ Second, our results can be used to identify the patients at highest risk of HAC or death during their admission based on their glucose levels and diabetes status. While we acknowledge that the relationship between peak glucose and adverse outcome may not necessarily be causal in nature, notification of day 2 peak glucose above a threshold at least identifies patients who are at increased risk of HAC and who may require specific interventions. Lastly, although current recommended BG targets for inpatients are agnostic to the presence or absence of diabetes, our results strengthen the hypothesis that the relationship between peak glucose and adverse outcomes is different in patients with and without diabetes^28^ and lend support to the establishment of more personalised glucose targets for the management of hyperglycaemia in hospital.

### Limitations

There are several limitations to this study, which was retrospective and observational. We cannot ascertain whether day 2 hyperglycaemia reflects more severe illness or is causal to worse outcomes.^10^ Although we adjusted for baseline differences, unintended bias is still likely to exist. HbA1c results were not available for the whole cohort; therefore, we are unable to quantify stress-induced hyperglycaemia.^35^ Some of the non-diabetes cohorts were likely to have undiagnosed diabetes. As BG testing is only performed in selected patients without diabetes in our hospital, the non-diabetes cohort may not have been representative of all inpatients without diabetes and was enriched for admissions where BG monitoring is more likely, such as those receiving glucocorticoids or enteral/parenteral feeding. We do not have information on the use of drugs which may disrupt glycaemia, the use of intravenous glucose, or parenteral or enteral nutrition. We used POC BG testing rather than continuous subcutaneous glucose monitoring, which would be a more accurate measure of peak glucose and would provide more information on glucose variability. Our approach did not distinguish between fasting and random BG test times. We were unable to ascertain whether therapeutic interventions modified the described associations.^36^ We were unable to determine if outcomes varied according to type of diabetes, due to low numbers. Analysis using peak glucose meant that the impact of hypoglycaemia was not examined. We did not demonstrate a J-shaped relationship with increased risk of harm at low BG levels, possibly due to low numbers. We were unable to consider early warning scores to quantify the degree of acute illness.^37^

One strength of our study is that we present real-world data according to BG status in a relatively large number of heterogeneous admissions. We ensured there was temporal separation between the occurrence of hyperglycaemia and any hospital-acquired outcome, thus avoiding the limitation of reverse causality where worsening glycaemia is due to the adverse event itself. When assessing for differences in predictive ability of day 1 and day 2 glucose levels, prior studies^13 22^ had compared the effect size between baseline and subsequent glucose measurements, keeping both variables in the same model. However, due to high multicollinearity between day 1 and day 2 glucose levels, we used Akaike information criterion as a method of comparison.

The peak glucose level during the second 24 hours of hospital admission is a robust predictor of hospital adverse outcomes in patients with or without diabetes, and for patients without diabetes, was associated with an increased risk of in-hospital death. This suggests that day 2 peak blood glucose levels may be more indicative of patient trajectories and potential complications, providing valuable insights for the preparation of guidelines to tailor timely personalised glycaemic interventions and enhance patient outcomes.

## supplementary material

10.1136/bmjopen-2024-089652 online supplemental file 1

10.1136/bmjopen-2024-089652 online supplemental file 2

## Data Availability

No data are available.

## References

[1] Pasquel FJ, Lansang MC, Dhatariya K (2021). Management of diabetes and hyperglycaemia in the hospital. Lancet Diabetes Endocrinol.

[2] Dhatariya KK, Umpierrez G (2023). Gaps in our knowledge of managing inpatient dysglycaemia and diabetes in non-critically ill adults: A call for further research. Diabet Med.

[3] ElSayed NA, Aleppo G, Bannuru RR (2024). 16. Diabetes Care in the Hospital: Standards of Care in Diabetes-2024. Diabetes Care.

[4] Cheung NW, Li S, Ma G (2008). The relationship between admission blood glucose levels and hospital mortality. Diabetologia.

[5] Qian J, Kuang L, Che L (2020). Maximum blood glucose levels during hospitalisation to predict mortality in patients with acute coronary syndrome: a retrospective cohort study. BMJ Open.

[6] Chen P, Chen L, Zhao X (2021). The Association of Mean Plasma Glucose and In hospital Death Proportion: A Retrospective, Cohort Study of 162,169 In-Patient Data. Int J Endocrinol.

[7] Parappil A, Depczynski B, Collett P (2010). Effect of comorbid diabetes on length of stay and risk of death in patients admitted with acute exacerbations of COPD. Respirology.

[8] Dungan KM, Braithwaite SS, Preiser JC (2009). Stress hyperglycaemia. *Lancet*.

[9] Mifsud S, Schembri EL, Gruppetta M (2018). Stress-induced hyperglycaemia. Br J Hosp Med (Lond).

[10] Scheen M, Giraud R, Bendjelid K (2021). Stress hyperglycemia, cardiac glucotoxicity, and critically ill patient outcomes current clinical and pathophysiological evidence. Physiol Rep.

[11] Umpierrez GE, Smiley D, Jacobs S (2011). Randomized study of basal-bolus insulin therapy in the inpatient management of patients with type 2 diabetes undergoing general surgery (RABBIT 2 surgery). Diabetes Care.

[12] Klonoff DC, Messler JC, Umpierrez GE (2021). Association Between Achieving Inpatient Glycemic Control and Clinical Outcomes in Hospitalized Patients With COVID-19: A Multicenter, Retrospective Hospital-Based Analysis. Diabetes Care.

[13] Mowery NT, Gunter OL, Dossett LA (2011). Failure to achieve euglycemia despite aggressive insulin control signals abnormal physiologic response to trauma. J Crit Care.

[14] Kim SH, Park KN, Choi SP (2015). Time to reach target glucose level and outcome after cardiac arrest patients treated with therapeutic hypothermia. J Crit Care.

[15] Kosiborod M, Inzucchi SE, Krumholz HM (2008). Glucometrics in patients hospitalized with acute myocardial infarction: defining the optimal outcomes-based measure of risk. Circulation.

[16] (2017). Australian Government.

[17] Charlson ME, Pompei P, Ales KL (1987). A new method of classifying prognostic comorbidity in longitudinal studies: development and validation. J Chronic Dis.

[18] Levey AS, Stevens LA, Schmid CH (2009). A new equation to estimate glomerular filtration rate. Ann Intern Med.

[19] Elm E von, Altman DG, Egger M (2007). Strengthening the reporting of observational studies in epidemiology (STROBE) statement: guidelines for reporting observational studies. *BMJ*.

[20] RCoreTeam (2023). R: A language and environment for statistical computing. R Foundation for Statistical Computing Vienna, Austria.

[21] Thouy F, Bohé J, Souweine B (2022). Impact of prolonged requirement for insulin on 90-day mortality in critically ill patients without previous diabetic treatments: a post hoc analysis of the CONTROLING randomized control trial. Crit Care.

[22] Goyal A, Mahaffey KW, Garg J (2006). Prognostic significance of the change in glucose level in the first 24 h after acute myocardial infarction: results from the CARDINAL study. Eur Heart J.

[23] Bar-Or D, Rael LT, Madayag RM (2019). Stress Hyperglycemia in Critically Ill Patients: Insight Into Possible Molecular Pathways. Front Med (Lausanne).

[24] Umpierrez GE, Isaacs SD, Bazargan N (2002). Hyperglycemia: an independent marker of in-hospital mortality in patients with undiagnosed diabetes. J Clin Endocrinol Metab.

[25] Holt RIG, Cockram CS, Ma RCW (2024). Diabetes and infection: review of the epidemiology, mechanisms and principles of treatment. Diabetologia.

[26] Depczynski B, Ta B, Lau SM (2024). Enhanced recovery after surgery: an update for the generalist. Med J Aust.

[27] Grealish M, Chiew AL, Varndell W (2021). The relationship between admission glucose and lactate with critical illness amongst adult patients presenting to the emergency department. Acta Diabetol.

[28] Krinsley JS, Brownlee M, Schwartz MW (2022). Blood glucose targets in the critically ill: is one size fits all still appropriate?. Lancet Diabetes Endocrinol.

[29] Honarmand K, Sirimaturos M, Hirshberg EL (2024). Society of Critical Care Medicine Guidelines on Glycemic Control for Critically Ill Children and Adults 2024. Crit Care Med.

[30] Ta B, Depczynski B, Ericksson W (2023). Decreased rates of hospital-acquired infection after introduction of an active surveillance, virtual glucose management system. Diabetes Res Clin Pract.

[31] Norris T, Razieh C, Yates T (2022). Admission Blood Glucose Level and Its Association With Cardiovascular and Renal Complications in Patients Hospitalized With COVID-19. Diabetes Care.

[32] Lazzeri C, Valente S, Chiostri M (2015). Early glucose variability in cardiogenic shock following acute myocardial infarction: a pilot study. Ther Adv Cardiovasc Dis.

[33] Lazzeri C, Bonizzoli M, Cianchi G (2020). The prognostic role of peak glycemia and glucose variability in trauma: a single-center investigation. Acta Diabetol.

[34] Engle K, Bacani G, Cook CB (2023). Glucometrics: Where Are We Now?. Curr Diab Rep.

[35] Roberts GW, Larwood C, Krinsley JS (2022). Quantification of stress-induced hyperglycaemia associated with key diagnostic categories using the stress hyperglycaemia ratio. Diabet Med.

[36] Depczynski B, Kamalakkannan A, Siklosi B (2024). Association between continued metformin use during hospital admission and hospital-acquired complications. Diabet Med.

[37] Olsen MT, Klarskov CK, Hansen KB (2024). Risk factors at admission of in-hospital dysglycemia, mortality, and readmissions in patients with type 2 diabetes and pneumonia. J Diabetes Complications.

