# Supplementary material for: Retrospective observational study of the association of peak blood glucose during the second 24 hours of admission with hospital-acquired complications in non-critical care admissions to a tertiary referral teaching hospital
Source: BMJ Open. 2025 Jan 14;15(1):e089652. doi: 10.1136/bmjopen-2024-089652 (PMC11752049; doi:10.1136/bmjopen-2024-089652)
Supplement: online supplemental file 1 [file bmjopen-15-1-s001.docx]

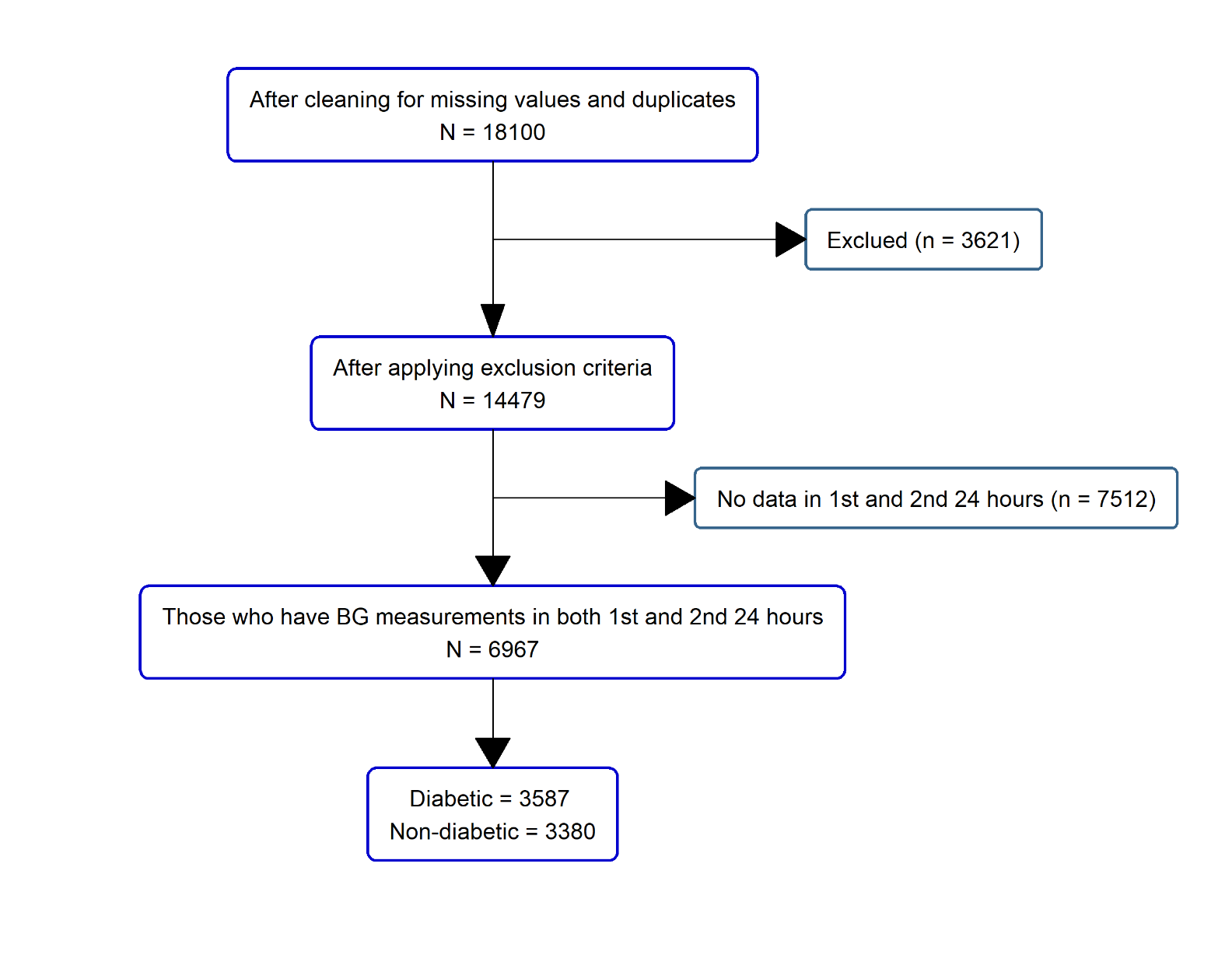


Supplementary Figure 1: Sampling flow chart for hospital acquired complications and in hospital mortality, 1 January 2018 to 28 February 2021.
