# Supplementary material for: Retrospective observational study of the association of peak blood glucose during the second 24 hours of admission with hospital-acquired complications in non-critical care admissions to a tertiary referral teaching hospital
Source: BMJ Open. 2025 Jan 14;15(1):e089652. doi: 10.1136/bmjopen-2024-089652 (PMC11752049; doi:10.1136/bmjopen-2024-089652)
Supplement: online supplemental file 2 [file bmjopen-15-1-s002.docx]

Supplementary Table 1: Comparison of adjusted HRs and the Akaike information criterion (AIC) to compare the influence of peak glucose levels in the two different periods.

| Outcome | HR_First_24_Hours | Lower_CI_First_24 | Upper_CI_First_24 | HR_Second_24_Hours | Lower_CI_Second_24 | Upper_CI_Second_24 | AIC_First_24_Hours | AIC_Second_24_Hours | AIC_diff |
| --- | --- | --- | --- | --- | --- | --- | --- | --- | --- |
| HAC-Any | 1.039 | 1.025 | 1.053 | 1.057 | 1.041 | 1.073 | 16163.781 | 16125.261 | -38.520 |
| HAC-Infection | 1.021 | 1.003 | 1.040 | 1.030 | 1.0009 | 1.052 | 7042.280 | 7037.068 | -5.212 |
| HAC-Cardiac | 1.029 | 1.001 | 1.059 | 1.044 | 1.012 | 1.078 | 2840.284 | 2834.535 | -5.749 |
| HAC-In-Hospital Mortality | 1.0003 | 0.975 | 1.032 | 0.975 | 0.940 | 1.012 | 3465.713 | 3468.430 | 2.717 |
