# Supplementary material for: Retrospective observational study of the association of peak blood glucose during the second 24 hours of admission with hospital-acquired complications in non-critical care admissions to a tertiary referral teaching hospital
Source: BMJ Open. 2025 Jan 14;15(1):e089652. doi: 10.1136/bmjopen-2024-089652 (PMC11752049; doi:10.1136/bmjopen-2024-089652)
Supplement: Supplementary file 3 [file bmjopen-2024-089652.reviewer_comments.pdf]

PEER REVIEW HISTORY

BMJ Open publishes all reviews undertaken for accepted manuscripts. Reviewers are asked to complete a checklist review form and are provided with free text boxes to elaborate on their assessment. These free text comments are reproduced below.

ARTICLE DETAILS

Title (Provisional)

Retrospective Observational Study of the Association of Peak Blood Glucose during the Second 24 hours of Admission with Hospital-Acquired Complications in Non-Critical Care Patients at a Tertiary Referral Teaching Hospital.

Authors

Depczynski, Barbara; Kibret, Getiye Dejenu; Georgiou, A; Lau, Sue Mei

---

VERSION 1 - REVIEW

**Reviewer**	**1**
**Name**	**Olsen, Mikkel**
**Affiliation**	**Copenhagen University Hospital**
**Date**	**30-Jul-2024**
**COI**	**None.**

The authors performed a retrospective observational study of patients

admitted to non-critical care. The aim was to determine the relationship between the day 2 peak blood glucose and the occurrence of hospital-acquired complications or

in-hospital mortality. I want to congratulate the authors with the study.

I have comments below:

Introduction:

Regarding the guidelines listed, I recommend to add a that the in-hospital glycaemic target of approx 5-10 mmol/l accounts for "most hospitalised patients" (i.e., not necessarily terminal patients etc.).

In the summary, page 4: "We have identified the important need to explore personalized glycemic targets in non-critical care admission" I disagree that this is the objective of the study and that authors have done this. In stead, one could focus on the importance of reducing in-hospital hyperglycaemia to reduce adverse outcomes

In line with this, the sentence in the introduction: “In determining glycemic thresholds for intervention, assessment of day 2 glycaemia may provide a truer indicator of the quality of

diabetes care and associated benefits11,1" the threshold for when adverse outcomes are occurring does not necessarily determine the threshold for (insulin) intervention, I would argue, especially not for planning basal or prandial insulin in an in-hospital setting.

Aim:

You state that you want to determine the relationship between the peak BG observed during day 2 of admission and the subsequent occurrence of hospital acquired complications (HACs) or in-hospital mortality in patients with and without diabetes on non-ICU wards. In the statistics you also mention that you examine day 1 glycaemia line 25 page 8: "We examined how day 1 and day 2 peak glucose levels affect HACs by fitting these variables into separate models while controlling for the same set of confounders in both analyses"

Methods:

Setting: Are patients managed primarily by correctional insulin/sliding scale insulin, a basal-bolus insulin regimen, or others? Continuation of non-insulin antidiabetics during admission?

Definitions: why were the scores for diabetes omitted in the charlson comorbidity index?

Statistical analysis: In the work by Olsen et al." Risk factors at admission of in-hospital dysglycemia, mortality, and readmissions in patients with type 2 diabetes and pneumonia", the risk of in-hospital mortality increased with the degree of acute illness (i.e., high EWS) at admission which in adjusted models overruled the influence of in-hospital (dys)glycaemia. Do you have access to the EWS/Early warning scores? Otherwise, this could be discussed in the limitations.

Results

For the baseline analysis, how many had type 1 vs type 2 diabetes? Did the outcomes differ between the subgroups of diabetes?

You have examined hyperglycaemia´s relevance (peak gluocose level at day 2) for adverse outcomes. Have you data on glycaemic variability and hypoglycamia?

Figures 1 and 2: Hard to read due to small fond size.

Discussion

Line 30, page 13: "Secondly, our results can be used to inform appropriate glucose thresholds for electronic notification systems or direct management by the diabetes team" As previously noted, I disagree with this sentence. We need more data on this before a statement like this is true. E.g. rising data indicates that glycaemic targets for inpatients should be based on their out-patient glycaemic status, i.e. HbA1c. See e.g. Krinsley et al "The interaction of acute and chronic Glycemia on the relationship of hyperglycemia, hypoglycemia, and glucose variability to mortality in the critically ill"

Limitations:

I would recommend to include a statement that the identified peak glucose levels is perhaps not always truly identified since you used point-of-care glucose testing as glucose assessment tool and not continuous glucose monitoring.

Reviewer **2**

Name **SALINERO-FORT, MIGUEL**

Affiliation **Foundation for Biomedical Research and Innovation in Primary Care of the Community of Madrid**

Date **29-Sep-2024**

COI **I have no conflicts of interest**

The authors aimed to evaluate the impact of point-of-care peak capillary glucose values observed during the second day of hospitalization on hospital-acquired complications (HACs) or in-hospital mortality in patients with and without diabetes in non-ICU wards.

This research is conducted using a robust methodology to ensure the reliability of the findings. The study's objective, design, and statistical analysis are sound, and the discussion is appropriate

However, there are a few points that need to be addressed before the manuscript can be published.

It is not specified whether glycemic measurements are based on fasting glucose or opportunistic glycemia in patients without diabetes. During admission, these patients may require vasoactive drugs as well as glucose solution, so glycemia after these treatments is influenced by their non-basal nature. Additionally, in patients without diabetes, only a determination of capillary glucose level may have been made. It would be necessary to know the number of glucose values per patient on the second day to address this issue.

The second through fourth quartiles of peak glucose values have the highest variability. It is puzzling why the second quartile has the highest "glucose level (mean)", the highest patient age, and the highest incidence of HAC, HAC cardiology, and HAC infection. A reasonable explanation is needed, especially since the second quartile is unlikely to have the highest "glucose level (mean)" because an extreme value in the fourth quartile would skew the average upward and have the highest mean. Additionally, in Table 1, the values for "Glucose Level (Mean)" [11.2 mmol/l] and "Mean glucose day 2" [11.5 mmol/l] do not match. The "Glucose Level (Mean)" also has the same value as "Mean Glucose Day 1".

VERSION 1 - AUTHOR RESPONSE

Reviewer: 1
Dr. Mikkel Olsen, Copenhagen University Hospital
Comments to the Author:
The authors performed a retrospective observational study of patients admitted to non-critical care. The aim was to determine the relationship between the day 2 peak blood glucose and the occurrence of hospital-acquired complications or in-hospital mortality. I want to congratulate the authors with the study.

I have comments below:

Introduction:
Regarding the guidelines listed, I recommend to add a that the in-hospital glycaemic target of approx 5-10 mmol/l accounts for "most hospitalised patients" (i.e., not necessarily terminal patients etc.).

The introduction has been revised according to Reviewer 1's recommendation:

Current guidelines from American Diabetes Association recommend for management of hyperglycemia in noncritical care settings, a glycemic goal of 5.6–10.0 mmol/L for most hospitalised patients, whether it is new hyperglycemia (newly diagnosed diabetes or stress hyperglycemia), or hyperglycemia related to diabetes prior to admission3. The Joint British Diabetes Societies for Inpatient Care group have recommended a blood glucose target range of 6·0–10·0 mmol/L for most inpatients with hyperglycemia, with an acceptable range of 4·0–12·0 mmol/L, with modification of lower acceptable glucose according to whether patient is prescribed insulin or insulin secretagogues2.

In the summary, page 4: "We have identified the important need to explore personalized glycemic targets in non-critical care admission" I disagree that this is the objective of the study and that authors have done this. In stead, one could focus on the importance of reducing in-hospital hyperglycaemia to reduce adverse outcomes

This section has been deleted.

~~We have identified the important need to explore personalized glycemic targets in non-critical care admission~~

In line with this, the sentence in the introduction: “In determining glycemic thresholds for intervention, assessment of day 2 glycaemia may provide a truer indicator of the quality of diabetes care and associated benefits11,1” the threshold for when adverse outcomes are occurring does not necessarily determine the threshold for (insulin) intervention, I would argue, especially not for planning basal or prandial insulin in an in-hospital setting.

We agree with Reviewer 1 and acknowledge that the glucose thresholds for adverse outcomes do not necessarily determine the threshold for insulin intervention in a hospital setting. The paragraph containing the sentence has been modified:

In assessing glycaemia and hospital outcomes ~~to inform glucose targets~~, many studies have focused on at admission glycaemia alone[4], or whole of admission mean glycaemia[5-7]. Hyperglycemia within the first 24 hours of admission is likely a reflection of stress hyperglycemia and prior glycemic control[8,9]. Whole of admission glycaemia may be influenced by the bidirectional relationship between glycaemia and adverse hospital events[10]. ~~In determining glycemic thresholds for intervention, a~~Assessment of day 2 glycaemia may provide a truer indicator of the quality of diabetes care and associated benefits[11,12] <u>although we acknowledge that the glucose threshold for adverse outcomes does not necessarily determine the threshold for insulin intervention in a hospital setting</u>. In intensive care units (ICU), earlier achievement of glycemic control is associated with better outcomes[13,14]. In patients admitted for COVID-19 infection, hyperglycemia after admission, (but not admission hyperglycemia) was a strong predictor of death among non-ICU patients[12]. Similarly, mean glycaemia during the first 48 hours of admission for acute myocardial infarction was a better predictor of mortality than admission glucose[15]. To date, however, a detailed understanding of the association of day 2 glycaemia with hospital outcomes in a heterogeneous group of medical or surgical patients admitted to non-ICU wards is lacking.

Aim:
You state that you want to determine the relationship between the peak BG observed during day 2 of admission and the subsequent occurrence of hospital acquired complications (HACs) or in-hospital mortality in patients with and without diabetes on non-ICU wards. In the statistics you also mention that you examine day 1 glycaemia line 25 page 8: “We examined how day 1 and day 2 peak glucose levels affect HACs by fitting these variables into separate models while controlling for the same set of confounders in both analyses”

The secondary objective of the study has been added to the aims section:

<u>A secondary aim is to compare the association of peak glucose levels in day 1 and day 2 with the risk for HAC.</u>

Methods:
Setting: Are patients managed primarily by correctional insulin/sliding scale insulin, a basal-bolus insulin regimen, or others? Continuation of non-insulin antidiabetics during admission?

This information has been added to the methods:

Inpatient care of diabetes is primarily the responsibility of the admitting team. Consultations to the diabetes service are made on an ad hoc basis. There are policies for management of hypoglycaemia and perioperative care of patients with diabetes. <u>Use of non-insulin anti-hyperglycaemic therapies are not prohibited and there is no standing order to discontinue oral anti-hyperglycaemic agents and routinely prescribe basal-bolus insulin regimen during the admission.</u>

Definitions: why were the scores for diabetes omitted in the charlson comorbidity index?

Omission of the scores for diabetes, is as per prior publications, eg

Depczynski B, Kamalakkannan A, Siklosi B, Lau SM. Association between continued metformin use during hospital admission and hospital-acquired complications. Diabet Med. 2024 Oct;41(10):e15353. doi: 10.1111/dme.15353. Epub 2024 May 31. PMID: 38820128.

Kyi M, Colman PG, Wraight PR, Reid J, Gorelik A, Galligan A, Kumar S, Rowan LM, Marley KA, Nankervis AJ, Russell DM, Fourlanos S. Early Intervention for Diabetes in Medical and Surgical Inpatients Decreases Hyperglycemia and Hospital-Acquired Infections: A Cluster Randomized Trial. Diabetes Care. 2019 May;42(5):832-840. doi: 10.2337/dc18-2342. Epub 2019 Mar 28. PMID: 30923164.

We exclude diabetes from the Charlson comorbidity index calculation because we used diabetes status as a separate variable and diabetes would be represented twice in the model—first as part of the CCI score and then as an individual variable—resulting in redundant information that could distort the relationship between predictors and the outcome.

Excluding diabetes from CCI calculation from the diabetes only analyses helps in isolating the effect of hyperglycaemia by adjusting for comorbidities unrelated to diabetes, giving a clearer view of non-diabetes-related health burdens.

Maintaining the CCI calculation without diabetes across analyses promotes consistency and helps avoid multicollinearity, ensuring comparability of the impact of day 2 hyperglycemia across population groups with consistent handling of confounders.

Statistical analysis: In the work by Olsen et al." Risk factors at admission of in-hospital

dysglycemia, mortality, and readmissions in patients with type 2 diabetes and pneumonia", the risk of in-hospital mortality increased with the degree of acute illness (i.e., high EWS) at admission which in adjusted models overruled the influence of in-hospital (dys)glycaemia. Do you have access to the EWS/Early warning scores? Otherwise, this could be discussed in the limitations.

We do not have access to early warning score, and so this limitation has been added to the limitations section.

We were unable to consider early warning scores to quantify degree of acute illness (Olsen 2024).

Results
For the baseline analysis, how many had type 1 vs type 2 diabetes? Did the outcomes differ between the subgroups of diabetes?

We did not undertake a diabetes subgroup analysis as the number of patients with T1DM was low, 1.7% of the diabetes cohort. as given in the methods, admissions for

hyperglycemic and hypoglycemic emergencies were excluded. The results and limitations section has been altered accordingly.

<u>In results</u>

Of the whole cohort, 3930 (56.4%) were men and 3587(51.5%) had a diagnosis of diabetes, <u>(type 1 in 61 patients).</u>

<u>In limitations</u>

<u>We were unable to determine if outcomes varied according to type of diabetes, due to low numbers</u>.

You have examined hyperglycaemia´s relevance (peak gluocose level at day 2) for adverse outcomes. Have you data on glycaemic variability and hypoglycamia?

We agree with Reviewer 1 that this type of analysis would be of great interest, but such an analysis is outside the scope of our manuscript. Our discussion already acknowledged that* "analysis using peak glucose meant that the impact of hypoglycemia was not examined". *As Reviewer 1 has commented below a limitation is the analysis has been performed using POC BG tests rather than with continuous glucose monitoring which is currently not widely available in the hospital setting.

The following has been added to the limitation section:

<u>We used POC BG testing rather than continuous subcutaneous glucose monitoring, which would be a more accurate measure of peak glucose and would provide more information on glucose variability.</u>

Figures 1 and 2: Hard to read due to small fond size.

The figures have been revised to improve readability.

Discussion
Line 30, page 13: "Secondly, our results can be used to inform appropriate glucose thresholds for electronic notification systems or direct management by the diabetes team" As previously noted, I disagree with this sentence. We need more data on this before a statement like this is true. E.g. rising data indicates that glycaemic targets for inpatients should be based on their out-patient glycaemic status, i.e. HbA1c. See e.g. Krinsley et al "The interaction of acute and chronic Glycemia on the relationship of hyperglycemia, hypoglycemia, and glucose variability to mortality in the critically ill"

We acknowledge Reviewer 1's comments. The statement has been deleted and revised:

~~Secondly, our results can be used to inform appropriate glucose thresholds for electronic notification systems or direct management by the diabetes team.~~ Secondly, our results can be used to identify the patients at highest risk of HAC or death during their admission, based on their glucose levels and diabetes status.

Limitations:

I would recommend to include a statement that the identified peak glucose levels is perhaps not always truly identified since you used point-of-care glucose testing as glucose assessment tool and not continuous glucose monitoring.

We agree with Reviewer 1 and as discussed above have added the following to the limitations:

A limitation is that we used POC BG testing rather than continuous subcutaneous glucose monitoring, which would be a more accurate measure of peak glucose and would provide more information on glucose variability.

Reviewer: 2
Dr. MIGUEL SALINERO-FORT, Foundation for Biomedical Research and Innovation in Primary Care of the Community of Madrid, IdiPAZ
Comments to the Author:
The authors aimed to evaluate the impact of point-of-care peak capillary glucose values observed during the second day of hospitalization on hospital-acquired complications (HACs) or in-hospital mortality in patients with and without diabetes in non-ICU wards.
This research is conducted using a robust methodology to ensure the reliability of the findings.
The study's objective, design, and statistical analysis are sound, and the discussion is appropriate
However, there are a few points that need to be addressed before the manuscript can be published.
It is not specified whether glycemic measurements are based on fasting glucose or opportunistic glycemia in patients without diabetes.

Specification of type of glycemic measurement in patients without diabetes has been added to the methods section:

Opportunistic POC BG testing is encouraged for those without known diabetes at risk for hyperglycemia, for example patients on stroke and cardiac wards, or those on glucocorticoids.

During admission, these patients may require vasoactive drugs as well as glucose solution, so glycemia after these treatments is influenced by their non-basal nature.

The following has been added to the limitations:

<u>We do not have information on use of drugs which may disrupt glycaemia, use of intravenous glucose, or parenteral or enteral nutrition</u>.

Additionally, in patients without diabetes, only a determination of capillary glucose level may have been made. It would be necessary to know the number of glucose values per patient on the second day to address this issue.

We agree with Reviewer 2 and the following has been added to the results section:

<u>The mean (SD) number of BG measurements per person during day 2 was 3.3 (2.3) in the overall group with 4.2 (2.4) in the diabetes patients and 2.24 (1.6) in the non-diabetes patients</u>.

The second through fourth quartiles of peak glucose values have the highest variability. It is puzzling why the second quartile has the highest "glucose level (mean)", the highest patient age, and the highest incidence of HAC, HAC cardiology, and HAC infection. A reasonable explanation is needed, especially since the second quartile is unlikely to have the highest "glucose level (mean)" because an extreme value in the fourth quartile would skew the average upward and have the highest mean.

I apologise to Reviewer 2, as the labels for the quartiles were switched when importing from Excel to Word.

The corrected Table 1 is now given below.

Additionally, in Table 1, the values for "Glucose Level (Mean)" [11.2 mmol/l] and "Mean glucose day 2" [11.5 mmol/l] do not match. The "Glucose Level (Mean)" also has the same value as "Mean Glucose Day 1".

The row "Glucose level mean (SD)" is the mean glucose of day 1. The row has been deleted due to duplication.

Table 1

**Variable**	**All**	**Diabetes**	**No Diabetes**	**≤6.7mmol/L**	**6.8-8.3mmol/L**	**8.4<11mmol/L**	**≥11mmol/L**
Patients, n	6967	3587	3380	1840	1746	1672	1709
Gender, n (%)							
Female	3037 (43.6)	1463 (40.8)	1574 (46.6)	881 (47.9)	769 (44)	683 (40.8)	704 (41.2)
Male	3930 (56.4)	2124 (59.2)	1806 (53.4)	959 (52.1)	977 (56)	989 (59.2)	1005 (58.8)
Age, median (Q1, Q3)	71 (59, 80)	74 (65, 81)	67 (52, 79)	69 (52, 80)	71 (58.25, 80)	72 (62, 81)	73 (63, 81)
Admission Type, n (%)							
Emergency	5295 (76)	2658 (74.1)	2637 (78)	1526 (82.9)	1300 (74.5)	1175 (70.3)	1294 (75.7)

Elective	1672 (24)	929 (25.9)	743 (22)	314 (17.1)	446 (25.5)	497 (29.7)	415 (24.3)
Admitting team, n (%)							
Medical	4171 (59.9)	2184 (60.9)	1987 (58.8)	1180 (64.1)	1016 (58.2)	943 (56.4)	1032 (60.4)
Surgical	2796 (40.1)	1403 (39.1)	1393 (41.2)	660 (35.9)	730 (41.8)	729 (43.6)	677 (39.6)
LOS, median (Q1, Q3)	6 (4, 12)	6 (4, 11)	6 (4, 12)	6 (3, 11)	6.5 (4, 13)	7 (4, 13)	6 (4, 12)
Hospital Mortality, n (%)	228 (3.3)	89 (2.5)	139 (4.1)	56 (3)	62 (3.6)	64 (3.8)	46 (2.7)
HAC Types, n (%)							
HAC Any	930 (13.3)	492 (13.7)	438 (13)	146 (7.9)	228 (13.1)	257 (15.4)	299 (17.5)
HAC Infection	493 (7.1)	259 (7.2)	234 (6.9)	59 (3.2)	116 (6.6)	150 (9)	168 (9.8)
HAC Cardiac	208 (3)	127 (3.5)	81 (2.4)	27 (1.5)	47 (2.7)	47 (2.8)	87 (5.1)
Charlson Index, n (%)							
None	4760 (68.3)	2252 (62.8)	2508 (74.2)	1347 (73.2)	1223 (70)	1135 (67.9)	1055 (61.7)
Mild	1388 (19.9)	785 (21.9)	603 (17.8)	329 (17.9)	344 (19.7)	345 (20.6)	370 (21.7)
Moderate	508 (7.3)	351 (9.8)	157 (4.6)	91 (4.9)	111 (6.4)	125 (7.5)	181 (10.6)
Severe	311 (4.5)	199 (5.5)	112 (3.3)	73 (4)	68 (3.9)	67 (4)	103 (6)
Mean glucose day 1, mean (SD)	8.2 (2.9)	9.5 (3.3)	6.9 (1.6)	6.5 (1.3)	7.2 (1.6)	8.2 (2.1)	11.2 (3.5)
Mean glucose day 2, mean (SD)	8 (2.6)	9.1 (2.9)	6.9 (1.6)	5.7 (0.6)	6.9 (0.6)	8.3 (1)	11.5 (2.7)
HbA1c, mean (SD)<br>N=number of patients with result	7.5 (2)	7.9 (2)<br>N=1955	5.7 (0.6)<br>N=492	5.8 (0.7)	6.3 (1.1)	7.2 (1.5)	8.6 (2.1)
DM therapies							
Insulin (with or without other anti-hyperglycaemic therapy)	838 (12)	838 (23.4)	0 (0)	6 (0.3)	59 (3.4)	187 (11.2)	586 (34.3)
Any non-insulin antihyperglycemic therapy	2253 (32.3)	2253 (62.8)	0 (0)	271 (14.7)	496 (28.4)	680 (40.7)	806 (47.2)
No antihyperglycemic therapy	3876 (55.6)	496 (13.8)	3380 (100)	1563 (84.9)	1191 (68.2)	805 (48.1)	317 (18.5)
Glucocorticoids during 1st 24 hr	990 (14.2)	334 (9.3)	656 (19.4)	205 (11.1)	240 (13.7)	258 (15.4)	287 (16.8)
Admission eGFR, mean (SD)	73.3 (29.1)	66.5 (28.4)	80.6 (28.1)	78.4 (28.7)	75.8 (27.4)	71.7 (28.9)	66.9 (30)
Year, n (%)							
2018	2561 (36.8)	1474 (41.1)	1087 (32.2)	619 (33.6)	622 (35.6)	617 (36.9)	703 (41.1)
2019	2225 (31.9)	1108 (30.9)	1117 (33)	606 (32.9)	558 (32)	539 (32.2)	522 (30.5)
2020 & 2021	2181 (31.3)	1005 (28)	1176 (34.8)	615 (33.4)	566 (32.4)	516 (30.9)	484 (28.3)

VERSION 2 - REVIEW

**Reviewer**	**1**
**Name**	**Olsen, Mikkel**
**Affiliation**	**Copenhagen University Hospital**
**Date**	**20-Nov-2024**

COI

The authors have nicely addressed all my concerns. Thank you for providing a clear point-by-point letter.

Reviewer 2

Name SALINERO-FORT, MIGUEL

Affiliation Foundation for Biomedical Research and Innovation in Primary Care of the Community of Madrid

Date 03-Dec-2024

COI

The authors satisfactorily answered all the issues raised, and the suggested changes have been implemented.
